# Isotropization of a Rotating and Longitudinally Expanding *ϕ*^4^ Scalar System

**DOI:** 10.3390/e24111612

**Published:** 2022-11-05

**Authors:** Margaret E. Carrington, Gabor Kunstatter, Christopher D. Phillips, Marcelo E. Rubio

**Affiliations:** 1 Department of Physics, Brandon University, Brandon, MB R7A 6A9, Canada; 2 Winnipeg Institute for Theoretical Physics, Winnipeg, MB R3T 2N2, Canada; 3 Department of Physics, University of Winnipeg, Winnipeg, MB R3M 2E9, Canada; 4 Department of Physics, Simon Fraser University, Burnaby, BC V5A 1S6, Canada

**Keywords:** isotropization, plasmas, angular momentum

## Abstract

We study numerically the evolution of an expanding system of scalar fields. The initial configuration is non-isotropic and rotating. We calculate the energy–momentum tensor and angular momentum vector of the system. We compare the time scales associated with the isotropization of the transverse and longitudinal pressures, and the decay of the initial angular momentum. We show that even a fairly large initial angular momentum decays significantly faster than the pressure anisotropy.

## 1. Introduction

In this paper, we study the time evolution of an expanding system of rotating massless real scalar fields with quartic coupling. Our calculation is based on the method developed in [1,2]. Observables calculated in a loop expansion exhibit divergences at next-to-leading order, which originate from instabilities in the classical solutions. The effect is seen in a calculation of the energy–momentum tensor at the next-to-leading order, where the energy density and pressures of the system diverge rapidly with increasing time. Gelis and his collaborators have shown that this problem can be cured using a resummation scheme that collects the leading secular terms at each order of an expansion in the coupling constant. This resummation can be performed by allowing the initial condition for the classical field to fluctuate, and averaging over these fluctuations. They have shown that a system of scalar fields isotropizes when this resummation is performed [2].

The motivation behind the development of this approach is to study the thermalization of the glasma phase of the matter created in a relativistic heavy ion collision. It is known that a hydrodynamic description, which is valid when the system is fairly close to thermal equilibrium, works well at very early times (∼1 fm/c). Approaches that are based on kinetic theory descriptions of the scattering of quasi-particles cannot explain this rapid thermalization. Another possibility that has been studied extensively is that the system is strongly coupled, even at very high energies. The proposal of Gelis et al. is that rapid thermalization could be achieved by a resummation of quantum fluctuations. The color glass condensate (CGC) effective theory provides a natural framework for this formulation [3,4,5]. At very early times, the system is best described as a system of strong classical fields that can be obtained from solutions of the Yang–Mills equation using a CGC approach. The spectrum of quantum fluctuations was derived in [6]. The success of the resummation method was demonstrated in [7], where the authors showed that pressure isotropiztion occurs in an SU(2) analog of QCD.

Our ultimate goal is to use the Gelis et al. approach to study the creation and evolution of angular momentum in a glasma. This is interesting in the context of recent proposals that the glasma is produced in a rapidly rotating state, which could be detected by looking for the polarization of produced hyperons. There have been calculations that predict very large values for the initial angular momentum of the system [8,9,10], but significant final state polarization effects have not been observed [11,12]. In this paper, we develop a formulation to calculate the angular momentum of a system of real scalar fields. We present preliminary results that indicate the angular momentum relaxes to a small value on a time scale significantly smaller than the time scale for pressure isotropization. If a similar result is obtained in a QCD glasma, it would be consistent with the observations in [11,12]. We also comment that a calculation of angular momentum in glasma was conducted in [13], using a CGC approach with a proper time expansion, and it was found also that large amounts of angular momentum were not produced.

Since computations in a gauge theory are considerably more complicated, we will work with a scalar theory. While it is true that QCD and scalar $\phi^{4}$ theory are different in many ways, they have important similarities in the context of this calculation because they both have unstable modes and are scale invariant at the classical level. In addition, we will mimic the kinematics of a relativistic nuclear collision by working in Milne coordinates with a rapidity independent background field. Milne coordinates are suitable because in a nuclear collision, there is a preferred spatial direction provided by the collision axis, and in the high energy limit, one expects invariance under Lorentz boosts in the *z*-direction.

This paper is organized as follows. In Section 2, we describe the method, and in Section 3, we formulate the calculation of the energy–momentum tensor and angular momentum. Some details of our numerical procedure are discussed in Section 4. In Section 5, we present our results, and in Section 6 we make some concluding remarks.

Throughout this paper, the spacetime is always taken to be Minkowski, with the signature (+,−,−,−). In addition to standard inertial coordinates (t,x,y,z), we also use Milne coordinates (τ,x,y,η), where τ is the proper time and η is the spacetime rapidity. Finally, we choose units such that $c=k_{B}=\hbar =1$, where *c* is the speed of light in a vacuum, $k_{B}$ is the Boltzmann constant, and *ℏ* is the Planck constant divided by 2π.

## 2. Formalism

### 2.1. Preliminaries

We consider a massless self-interacting real scalar field ϕ with quartic coupling. The Lagrangian density is given by

(1) $$\begin{array}{c} L=\frac{1}{2}\partial^{\mu }\phi \partial_{\mu }\phi -\frac{g^{2}}{4!}\phi^{4} \end{array}$$

where *g* is the coupling constant. To mimic the kinematics of a high energy nuclear collision, we work in Milne coordinates $\left(\tau ,\eta ,{\vec{x}}_{⊥}\right)$ with

$$\begin{array}{ccc} \tau & = & \sqrt{t^{2}-z^{2}}\\ \eta & = & \frac{1}{2}\ln\left(\frac{t+z}{t-z}\right)\;. \end{array}$$

Under a Lorentz boost in the *z*-direction, the proper time is unchanged and η is shifted by a constant. The metric in Milne coordinates is

(2) $$\begin{array}{c} g_{\mu \nu }dx^{\mu }dx^{\nu }=d\tau^{2}-\tau^{2}d\eta^{2}-dx^{2}-dy^{2}\;. \end{array}$$

**Figure 1:**
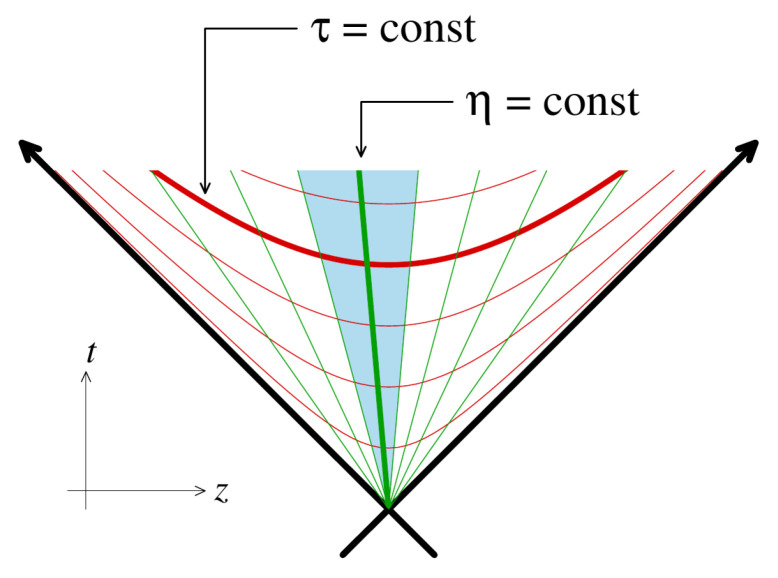
Representation of hypersurfaces of constant τ and η. The rapidity determines the location of a particle along a surface of fixed τ.

Figure 1 shows the curves of constant τ and η.

### 2.2. The Resummation Procedure

As explained in [1,2], observables calculated in a loop expansion exhibit secular divergences at next-to-leading order that originate from instabilities of the classical solutions. Gelis et al. proposed to cure this problem using a resummation scheme that collects the leading secular terms at each order of an expansion in the coupling constant, by averaging over an ensemble of initial conditions. The energy–momentum tensor is ultraviolet divergent, but the divergence corresponds to a vacuum contribution and can be removed by repeating the calculation with the background field set to zero, and subtracting the results. This vacuum subtraction was performed for all the calculations presented in this paper.

The equation of motion for the scalar field obtained from the Lagrangian (1) is

(3) $$\ddot{\phi }\left(\tau ,\eta ,{\vec{x}}_{⊥}\right)-\frac{1}{\tau }\dot{\phi }-\frac{1}{\tau^{2}}\partial_{\eta }^{2}\phi -\Delta_{⊥}\phi +\frac{g^{2}}{6}\phi^{3}=0$$

where the “dot” indicates a derivative with respect to τ, and $\Delta_{⊥}$ is the transverse Laplacian operator. The initial field is written as the sum of a background field contribution, φ, which is assumed to be boost invariant and therefore independent of η, and an η-dependent fluctuation, which we call α

(4) $$\begin{array}{c} \phi^{\chi }\left(\tau_{0},\eta ,{\vec{x}}_{⊥}\right)=\varphi \left(\tau_{0},{\vec{x}}_{⊥}\right)+\alpha^{\chi }\left(\tau_{0},\eta ,{\vec{x}}_{⊥}\right)\;. \end{array}$$

The initial time $\tau_{0}$ is chosen to be small but nonzero (see Section 4.3 for further discussion). The initial background field $\varphi (\tau_{0},{\vec{x}}_{⊥})$ is discussed in Section 4.5. The index χ in Equation (4) indicates that we have a Gaussian ensemble of initial conditions defined as

(5) $$\begin{array}{ccc} & & \alpha^{\chi }\left(\tau_{0},\eta ,{\vec{x}}_{⊥}\right)=\int dK\left[c_{K}^{\chi }a_{K}+c_{K}^{\chi \;*}a_{K}^{*}\right]\;. \end{array}$$

The index *K* labels the momentum variables $\left(\nu ,{\vec{k}}_{⊥}\right)$ that are conjugate to the coordinate-space variables $\left(\eta ,{\vec{x}}_{⊥}\right)$, respectively. The notation $c_{K}^{\chi }$ indicates an element in a Gaussian-distributed ensemble of $N_{\chi }$ random numbers, with variance

(6) $$\begin{array}{c} \langle c_{K}^{*}c_{L}\rangle =\frac{1}{2}\delta_{KL}\;. \end{array}$$

We use the momentum space integration measure

(7) $$\begin{array}{c} dK=\frac{d\nu }{2\pi }\frac{d{\vec{k}}_{⊥}}{{\left(2\pi \right)}^{2}} \end{array}$$

and the delta function in Equation (6) is defined so that $\int dK\delta_{KL}=1$. The mode functions $a_{K}\equiv a_{\nu {\vec{k}}_{⊥}}\left(\tau_{0},\eta ,{\vec{x}}_{⊥}\right)$ are obtained from the linearized equations of motion

(8) $$\begin{array}{c} {\ddot{a}}_{K}+\frac{1}{\tau }{\dot{a}}_{K}-\frac{1}{\tau^{2}}\partial_{\eta }^{2}a_{K}-\Delta_{⊥}a_{K}+\frac{g^{2}}{2}\varphi^{2}\left(\tau_{0},{\vec{x}}_{⊥}\right)a_{K}=0\; \end{array}$$

and normalized so that $\int dK(a_{K},a_{L})=1$ with

(9) $$\begin{array}{c} \left(a_{K},a_{L}\right)=i\tau \int d\eta \int d^{2}{\vec{x}}_{⊥}\;\left(a_{K}^{*}\partial_{\tau }a_{L}-\left(\partial_{\tau }a_{K}^{*}\right)a_{L}\right)\;. \end{array}$$

Separating variables and performing the normalization, one finds

(10) $$\begin{array}{ccc} a_{K} & \equiv & a_{\nu {\vec{k}}_{⊥}}\left(\tau_{0},\eta ,{\vec{x}}_{⊥}\right)=\frac{1}{2}\sqrt{\pi }e^{\pi \nu /2}\;e^{i\nu \eta }\chi_{{\vec{k}}_{⊥}}\left({\vec{x}}_{⊥}\right)H_{i\nu }^{\left(2\right)}\left(\lambda_{{\vec{k}}_{⊥}}\tau_{0}\right)\; \end{array}$$

where the $\chi_{{\vec{k}}_{⊥}}$ is the solution of the eigenvalue equation

(11) $$\begin{array}{c} \left[-\Delta_{⊥}+\frac{g^{2}}{2}\varphi^{2}\left(\tau_{0},{\vec{x}}_{⊥}\right)\right]\chi_{{\vec{k}}_{⊥}}\left({\vec{x}}_{⊥}\right)=\lambda_{{\vec{k}}_{⊥}}^{2}\chi_{{\vec{k}}_{⊥}}\left({\vec{x}}_{⊥}\right)\;. \end{array}$$

The field $\phi^{\chi }\left(\tau ,\eta ,{\vec{x}}_{⊥}\right)$ at finite proper time is obtained by solving Equation (3) with the initial condition $\phi^{\chi }\left(\tau_{0},\eta ,{\vec{x}}_{⊥}\right)$ obtained from Equations (4), (5)–(7), (10) and (11). From this point on, we drop the subscript χ.

## 3. Observables

### 3.1. Energy Momentum Tensor

The energy–momentum tensor of theory (1) is

(12) $$\begin{array}{c} T^{\mu \nu }=\partial^{\mu }\phi \partial^{\nu }\phi -g^{\mu \nu }\left[\frac{1}{2}\partial^{\alpha }\phi \partial_{\alpha }\phi -\frac{g^{2}}{4!}\phi^{4}\right]\;. \end{array}$$

The invariance of the Lagrangian under the conformal transformation

(13) $$\begin{array}{c} g_{\mu \nu }\to \Omega^{-2}g_{\mu \nu };\;\;\phi \to \Omega^{-1}\phi \end{array}$$

implies that $T^{\mu \nu }$ is traceless on shell.

The expressions for the energy and pressure are

(14) $$\begin{array}{c} \epsilon =T^{00}=\frac{1}{2}\left({\left(\partial_{\tau }\phi \right)}^{2}+\frac{{\left(\partial_{\eta }\phi \right)}^{2}}{\tau^{2}}+{\left(\partial_{x}\phi \right)}^{2}+{\left(\partial_{y}\phi \right)}^{2}\right)+V\left(\phi \right)\\ p_{L}=\tau^{2}T^{11}=\frac{1}{2}\left({\left(\partial_{\tau }\phi \right)}^{2}+\frac{{\left(\partial_{\eta }\phi \right)}^{2}}{\tau^{2}}-{\left(\partial_{x}\phi \right)}^{2}-{\left(\partial_{y}\phi \right)}^{2}\right)-V\left(\phi \right)\\ p_{T}=\frac{1}{2}\left(T^{22}+T^{33}\right)=\frac{1}{2}\left({\left(\partial_{\tau }\phi \right)}^{2}-\frac{{\left(\partial_{\eta }\phi \right)}^{2}}{\tau^{2}}\right)-V\left(\phi \right)\; \end{array}$$

where $V\left(\phi \right)=g^{2}\phi^{4}/4!$. In terms of the energy and pressure, the trace condition is

(15) $$\begin{array}{c} \epsilon =2p_{T}+p_{L}\;. \end{array}$$

### 3.2. Angular Momentum

We use the standard Pauli–Lubanski formalism [14,15] to obtain an expression for the angular momentum in terms of the energy–momentum tensor. We define the tensor field

(16) $$\begin{array}{c} M^{\mu \nu \lambda }=T^{\mu \nu }R^{\lambda }-T^{\mu \lambda }R^{\nu }\; \end{array}$$

where $R^{\mu }$ is the coordinate vector. Using Stokes’ theorem, one obtains a set of six conserved quantities

(17) $$\begin{array}{c} J^{\nu \lambda }=\int_{\Sigma }d^{3}y\sqrt{\left|\gamma \right|}\;n_{\mu }M^{\mu \nu \lambda }\;, \end{array}$$

where $n^{\mu }$ is a unit vector perpendicular to the hypersurface Σ, $\gamma_{ij}$ is the induced metric on this hypersurface, and $d^{3}y$ is the corresponding volume element. The angular momentum is obtained from the Pauli–Lubanski vector

(18) $$\begin{array}{c} L_{\mu }=-\frac{1}{2}\epsilon_{\mu \alpha \beta \rho }J^{\alpha \beta }u^{\rho } \end{array}$$

where $u^{\rho }$ is the vector that denotes the rest frame of the system. Equations (16)–(18) will give

(19) $$\begin{array}{c} L_{\mu }=-\frac{1}{2}\epsilon_{\mu \alpha \beta \rho }\int d^{3}y\sqrt{\gamma }\;n_{\sigma }\;u^{\rho }\;\left(T^{\sigma \alpha }R^{\beta }-T^{\sigma \beta }R^{\alpha }\right) \end{array}$$

where the energy–momentum tensor is given in Equation (12).

To find the angular momentum on a surface of constant τ, we define

(20) $$\begin{array}{c} n_{\mu }=\frac{\partial \tau }{\partial x^{\mu }}\;. \end{array}$$

In Minkowski coordinates, this gives $n_{\mu }=\left(\cos h\left(\eta \right),0,0,-\sin h\left(\eta \right)\right)$, and it is easy to verify that $n_{\mu }^{\mathrm{Milne}}=\left(1,0,0,0\right)$, as expected. The fluid velocity is the local rest frame in comoving coordinates, which is written $u_{\mathrm{Milne}}^{\rho }=\left(1,0,0,0\right)$. In Minkowski coordinates, this becomes $u^{\rho }=\left(\cos h\left(\eta \right),0,0,\sin h\left(\eta \right)\right)$. We could calculate the angular momentum directly in Minkowski coordinates, or alternatively, we could perform the calculation in Milne coordinates and perform a coordinate transformation to obtain the Minkowski space result. We checked our computations by verifying that both calculations give the same result. The components of the angular momenta about each of the Minkowski coordinate axes are

(21) $$\begin{array}{c} L_{t}=\tau \int d^{2}{\vec{x}}_{⊥}\;d\eta \;\sin h\left(\eta \right)\;\dot{\phi }\left(x\partial_{y}\phi -y\partial_{x}\phi \right)\\ L_{x}=\int d^{2}{\vec{x}}_{⊥}\;d\eta \;\dot{\phi }\;y\partial_{\eta }\phi \\ L_{y}=-\int d^{2}{\vec{x}}_{⊥}\;d\eta \;\dot{\phi }\;x\partial_{\eta }\phi \\ L_{z}=-\tau \;\int d^{2}{\vec{x}}_{⊥}\;d\eta \;\cos h\left(\eta \right)\;\dot{\phi }\;\left(y\partial_{x}\phi -x\partial_{y}\phi \right)\;. \end{array}$$

We note that all components of the angular momentum are dimensionless (in natural units, with ℏ=1).

## 4. Numerical Implementation

### 4.1. Lattice Discretization

We discretize in both directions in the transverse plane with *L* grid points and lattice spacing set to 1, which effectively means that we define all dimensionful quantities in terms of the transverse lattice grid spacing. The rapidity variable η is discretized with *N* grid points and lattice spacing *h*. We consider a unit slice of rapidity, and therefore take h=1/N.

The discretization of the transverse variables is straightforward. The discretized version of Equation (11) is

(22) $$\begin{array}{ccc} & & D_{ij;kl}\;\chi_{kl}=\lambda^{2}\chi_{ij} \end{array}$$

with

(23) $$\begin{array}{ccc} & & D_{ij;kl}=\left(4+V_{ij}^{\prime \prime }\right)\delta_{ik}\delta_{jl}-\left(\delta_{i+1\;k}+\delta_{i-1\;k}\right)\delta_{jl}-\delta_{ik}\left(\delta_{j+1\;l}+\delta_{j-1\;l}\right)\;. \end{array}$$

Since *D* is a rank 4 tensor with $L^{4}$ components, we obtain $L^{2}$ eigenfunctions $\chi_{ij}^{e}$, and *L* eigenvalues ${\left(\lambda^{2}\right)}^{e}$, with $e\in (1,L^{2})$. The normalized eigenfunctions are

(24) $$\begin{array}{c} \sum_{ij}\chi_{ij}^{*e}\chi_{ij}^{\bar{e}}=L^{2}\;\delta^{e\bar{e}} \end{array}$$

and the momentum integration is discretized as

(25) $$\begin{array}{c} \int \frac{d^{2}{\vec{k}}_{⊥}}{{\left(2\pi \right)}^{2}}\to \frac{1}{L^{2}}\sum_{e=1}^{L^{2}}\;. \end{array}$$

Since the spatial lattice spacing is set to 1, an integral over transverse coordinates is discretized as

(26) $$\begin{array}{c} \int d^{2}{\vec{x}}_{⊥}\to \sum_{i=0}^{L-1}\sum_{j=0}^{L-1}\;. \end{array}$$

The discretization of the longitudinal variables is a little more subtle. The constraint

(27) $$\begin{array}{c} \partial_{\eta }^{2}e^{i\nu \eta }=-\nu^{2}e^{i\nu \eta } \end{array}$$

gives

(28) $$\begin{array}{c} \varepsilon_{v}^{2}:=\nu^{2}={\left(\frac{2}{h}\sin\left(\frac{\pi v}{N}\right)\right)}^{2}\; \end{array}$$

and we replace $\nu \to \varepsilon_{v}$ in every factor $e^{\pi \nu /2}$ and in the Hankel functions. For the complex exponential, we use $e^{i\nu \eta }\to e^{\frac{2\pi ivn}{N}}$. The integral over ν becomes a sum over *v* using

(29) $$\begin{array}{c} \int \frac{d\nu }{2\pi }\to \frac{1}{Nh}\sum_{v=0}^{N-1}\;. \end{array}$$

Combining these expressions, we find the discretized versions of Equations (4), (6) and (10):

(30) $$\begin{array}{c} \alpha_{nij}\left(\tau \right)=\frac{1}{NL^{2}h}\sum_{v=0}^{N-1}\sum_{p=1}^{L^{2}}\left[c_{vp}a_{nij}^{vp}\left(\tau \right)\;+c.c.\right]\;\\ a_{nij}^{vp}\left(\tau \right)=\frac{1}{2}\sqrt{\pi }e^{\frac{2\pi ivn}{N}}\;\chi_{ij}^{p}\;e^{\pi \nu /2}H_{i\nu }^{\left(2\right)}\left(\lambda_{{\vec{k}}_{⊥}}\tau \right)\;\\ \langle c_{ve}c_{u\tilde{e}}^{*}\rangle =\frac{1}{2}NL^{2}h\delta_{vu}\delta_{e\tilde{e}}\;. \end{array}$$

To verify that discretization is performed correctly, we checked the discretized version of the normalization condition (9).

### 4.2. Boundary Conditions

We use periodic boundary conditions, which means that the indices (i,j) that correspond to the transverse spatial coordinates are defined as modulo *L*, and the index *n* for the rapidity is modulo *N*. The boundary conditions satisfy the self-adjointness condition

$$\begin{array}{ccc} & & \nabla_{F}\phi \left(x\right)=\phi \left(i+1\right)-\phi \left(i\right)\\ & & \nabla_{B}\phi \left(x\right)=\phi \left(i\right)-\phi \left(i-1\right)\\ & & \sum_{i}f\left(i\right)\left(\nabla_{F}g\left(i\right)\right)=-\sum_{i}\left(\nabla_{B}f\left(i\right)\right)g\left(i\right)\;. \end{array}$$

### 4.3. Hankel Functions

The differential equation for the mode function was solved by separating variables, which gives the solution in (10). The time-dependent part of the equation is of the second order, and has two independent solutions, which are the Hankel functions $H_{i\nu }^{\left(1\right)}\left(\lambda \tau \right)$ and $H_{i\nu }^{\left(2\right)}\left(\lambda \tau \right)$. We use only the second because it has positive frequency behavior at large times

(31) $$\begin{array}{c} \lim_{\tau \to \infty }H_{i\nu }^{\left(2\right)}\left(\tau \right)=\sqrt{\frac{2}{\pi \tau }}e^{-i(\tau -i\pi \nu /2-\pi /4)}\;. \end{array}$$

From now on, we suppress the superscript (2) on the Hankel function. When τ→0, the Hankel function oscillates like $e^{\pm i\tau \nu }$ and the derivative diverges. Numerically, we must start the evolution at a small positive time, which we choose as $\tau_{0}=10^{-2}$. One can check that the value chosen for this small initial time does not change the results at finite times.

We describe below our method to calculate the Hankel functions. First, we define the scaled function

(32) $$\begin{array}{c} h_{i\nu }\left(\lambda \tau \right)=e^{\pi \nu /2}H_{i\nu }\left(\lambda \tau \right) \end{array}$$

which is easier to calculate numerically. At large times, one can obtain the scaled Hankel function for given values of ν and λ from the asymptotic series

(33) $$\begin{array}{c} h_{i\nu }\left(\lambda \tau \right)=\sqrt{\frac{2}{\pi \lambda \tau }}e^{-i(\lambda \tau -\pi /4)}\sum_{k=0}^{n}t_{k}+O\left(\tau^{-(n+1)}\right)\\ t_{k}=\frac{{\left(-1\right)}^{k}}{k!{\left(2i\lambda \tau \right)}^{k}}\prod_{s=1}^{k}\left(\nu^{2}+\frac{{\left(2s-2\right)}^{2}}{4}\right)\;. \end{array}$$

This expression must be used carefully because the series does not converge for arbitrarily large values *n*. We proceed as follows. For a given value of ν and λ, choose some value of τ and look for a value of $k_{\max}$ so that $t_{k_{\max}+1}<10^{-9}$ and Max $\left(t_{k\leq k_{\max}}\right)<10^{6}$. If this $k_{\max}$ can be found, use Equation (33) with $n=k_{\max}$. If $k_{\max}$ does not exist, then increase the chosen value of τ and try again. Using this procedure, we can find $h_{i\nu }\left(\lambda \tau \right)$ and its first derivative for each value of ν and λ, for some (possibly very large) time. We then use adaptive fifth-order Runge–Kutta to find each Hankel function at the initial time $\tau_{0}$.

### 4.4. Discretized Derivatives

The conservation equation

(34) $$\begin{array}{c} \frac{\partial \epsilon }{\partial \tau }+\frac{\epsilon +p_{L}}{\tau }=0 \end{array}$$

is an exact equation that should be satisfied whether or not the system is in equilibrium. Additionally, we should have that the trace of the energy–momentum tensor is zero, so that Equation (15) is satisfied. It is easy to show analytically that these conditions are satisfied for background fields if we use forward derivatives: $\partial_{x}f\left(x\right)\to f\left(i+1\right)-f\left(i\right)$. The point is that while centered derivatives are not wrong, much larger lattices must be used to achieve the same numerical accuracy.

For angular momentum, the situation is different. All contributions to the angular momentum have an integral of the form $\int dx\;\dot{\varphi }\;\partial_{x}\phi$. If the initial value of $\dot{\varphi }$ is constant, the integrand is a total derivative and therefore the integral will give zero. However, this is not well satisfied numerically with forward derivatives. In the calculation of angular momentum, it is therefore better to use centered derivatives: $\partial_{x}f\left(x\right)\to \left(f\left(i+1\right)-f\left(i-1\right)\right)/2$.

### 4.5. Initial Conditions

The initial conditions that we use for the background field and its derivative are

(35) $$\begin{array}{c} \varphi \left(\tau_{0},i,j\right)=\varphi_{0}\;\cos\left(k_{x}i+k_{y}j\right)\;\\ \dot{\varphi }\left(\tau_{0},i,j\right)={\dot{\varphi }}_{0}\sin\left[\left(i-\frac{L+1}{2}\right)\frac{\pi }{L-1}\right]\;. \end{array}$$

The argument of the sine function is ∓π/2 at i=1 and i=L, and zero at i=(L+1)/2, so the field has negative ${\dot{\varphi }}_{0}$ on the left side of the lattice and positive ${\dot{\varphi }}_{0}$ on the right side.

The astute reader will note that our initial classical field is not periodic, and therefore does not respect our boundary conditions. The reason is that we wish to avoid problems that may arise when resonant modes are considered, which in the present model would correspond to the normal modes of the finite spatial lattice. For a large enough lattice, all modes are effectively periodic, and it is therefore expected that the precise form of the initialization is not important.

## 5. Results and Discussion

All of our results are obtained with L=41 spatial grid points, N=120 points for the rapidity coordinate, and $N_{\chi }=256$ configurations. The initial conditions for the background field are obtained from (35) with $\varphi_{0}=15$, $k_{x}=k_{y}=1/\sqrt{2}$ and ${\dot{\varphi }}_{0}=10$.

**Figure 2:**
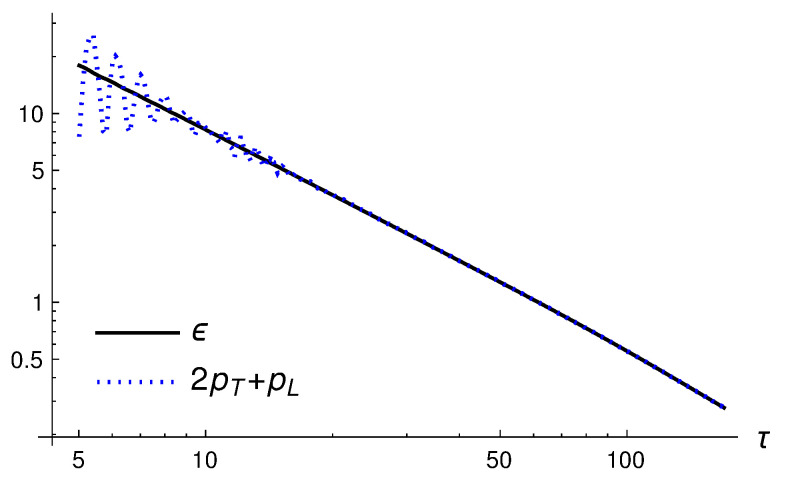
The energy and sum of the pressures as functions of τ.

To investigate if the system obeys Equation (15), we compare the energy density and the sum of the pressures. This is shown in Figure 2. One sees that after some initial oscillations have damped out, the condition $\epsilon =2p_{T}+p_{L}$ is well satisfied.

**Figure 3:**
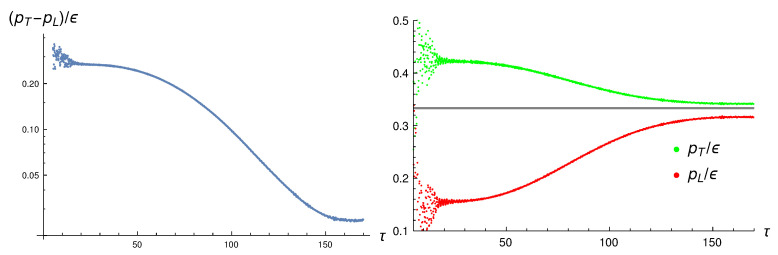
The transverse and longitudinal pressures, normalized by the energy density.

To see if the system approaches an isotropic state, and if it obeys an equation of state, we look at the transverse and longitudinal pressures. The left panel of Figure 3 shows that, after some initial oscillations have disappeared, the transverse and longitudinal pressures approach each other up to a time of about τ≈160. The right panel shows the two pressures normalized by the energy density, both approaching 1/3, again up to τ≈160. For large times, the simulation breaks down, which is not unexpected when one studies the dynamics of an expanding system inside a box of finite size.

**Figure 4:**
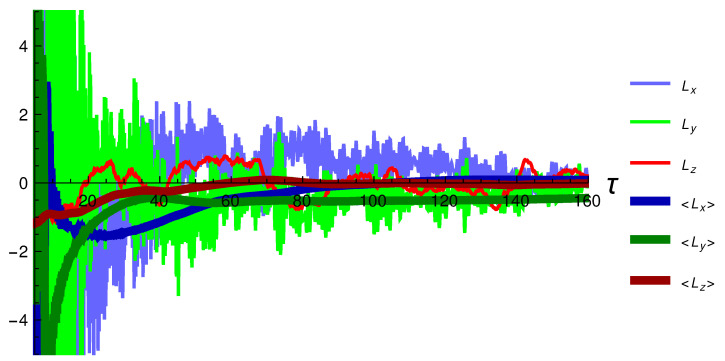
The three components of the angular momentum vector and their accumulated averages.

In Figure 4, we show the three components of the angular momentum in Equation (21). The *z* component, which depends weakly on the rapidity, is averaged over the unit slice of rapidity that we consider. In comparison with the energy and pressure, the oscillatory behavior is more severe and does not completely disappear. To obtain a better idea of the overall behavior, we also plot the accumulated average for each component, which is shown in Figure 4 with the thick lines. In each case, the darker color corresponds to the average of the component with the same but lighter color. The figure shows that even a fairly large initial angular momentum decays very quickly.

**Figure 5:**
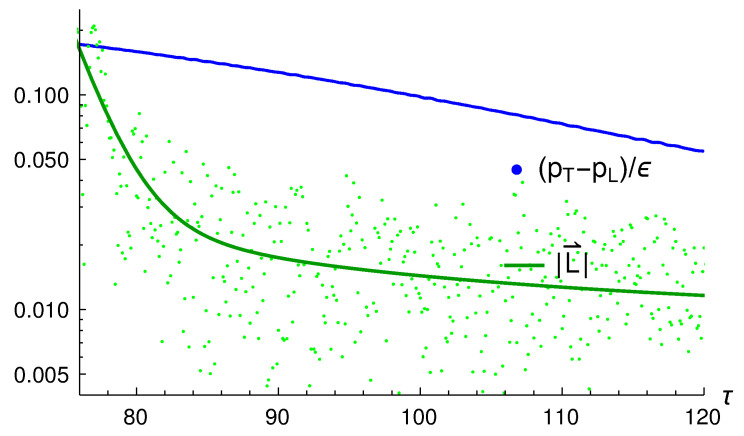
A comparison of $(p_{T}-p_{L})/\epsilon$ (blue) and $|\vec{L}|$ (green); see text for details.

We want to compare the time scales for the isotropization of the pressures, and the decay of the initial angular momentum. In Figure 5, we show in blue the curve in the left panel of Figure 3 over the range of τ for which the decay is strongest. To produce the light green points, we took the data for $|\vec{L}|$ versus τ with τ>12.0, where the large initial fluctuations are mostly gone, and shifted the first point (which was (12.0, 9.10)) so that it sits on top of the first point of the data that made the blue curve. The dark green line is a fit obtained for these data using the function $A+B/\tau +Ce^{-D\tau }$. The plot shows clearly that the initial angular momentum decays much more quickly than the pressure anisotropy, although the dispersion in the data is large. To quantify this dispersion, we calculated

(36) $$\begin{array}{c} \sigma =\frac{1}{N}\sum_{i=1}^{N}\frac{\sqrt{{\left(L_{i}-L_{\mathrm{fit}}\left(\tau_{i}\right)\right)}^{2}}}{\sqrt{{\left(L_{i}+L_{\mathrm{fit}}\left(\tau_{i}\right)\right)}^{2}}} \end{array}$$

where the sum is over the points shown in green in Figure 5 and $L_{\mathrm{fit}}\left(\tau \right)$ is the fitted function that gives the green curve. The result is σ=0.22.

## 6. Conclusions

In this paper, we presented some preliminary results from our study of the angular momentum in an expanding system of rotating massless scalar fields. Our results indicate that even when a large amount of angular momentum is put into the system, it decays very rapidly. Future work will include an investigation of how much these results depend on the exact form of the initialization and the boundary conditions, and possibly the extension of the calculation to physical theories, such as QCD. 

## Data Availability

Not applicable.
